# A Comparison of the Treatment of Periodontal Infraosseous Defects with or Without Biomaterials by a Minimally Invasive Surgical Approach

**DOI:** 10.3390/jcm14041111

**Published:** 2025-02-09

**Authors:** Alberto Gómez-Menchero, Marina Royón-Gálvez, Rodrigo Andrés-García, Mariano Herrero-Climent, Blanca Ríos-Carrasco, Fernando Fernandez-Palacín, Reyes Jaramillo-Santos

**Affiliations:** 1 Department of Periodontology, Dental School, University of Seville, 41009 Seville, Spain; alberto@gomezmenchero.com (A.G.-M.); marina.royon@gmail.com (M.R.-G.); mrjaramillo@us.es (R.J.-S.); 2 Department of surgery, Medical School, Universtity of Salamanca; Patio de Escuelas, 1, 37008 Salamanca, Spain; randresgarcia@gmail.com; 3 Porto Dental Institute, Periodontology Department, Symmetrya Prothesis, Av. de Montevideu 810, 4150-518 Porto, Portugal; dr.herrero@herrerocliment.com; 4 Departament of Stadistic and O.I., Faculty of Marine and Enviromental Science, University of Cadiz, 11003 Cadiz, Spain; fernando.fernandez@uca.es

**Keywords:** enamel matrix proteins, minimally invasive surgical procedure, periodontal defect, bone graft

## Abstract

**Background**: The aim of this study was to evaluate the clinical and radiological efficacy of minimally invasive surgical techniques for infraosseous defects by evaluating the effects of Emdogain (EMD) applied alone or with a xenograft, with a follow-up period of one year. **Methods**: Forty patients with a combined total of forty-eight intraosseous defects participated in the research. Of these, 20 participants were treated with EMD (group 1), and 28 were treated with EMD and a xenograft (group 2). The clinical measurements probing depth (PD), recession (REC), and clinical attachment level (CAL) were assessed at six sites on the tooth. Additionally, the following intraoperative clinical measurements were taken: (1) distance from the cementoenamel junction (CEJ) to the bottom of the defect (CEJ-BD) and (2) distance from the CEJ to the most coronal extension of the interproximal bone crest (CEJ-BC). The infraosseous component of the defect was defined as INTRA = (CEJ-BD) − (CEJ-BC). **Results**: In comparison with the baseline, both treatment options showed statistically significant improvements in reducing PD and increasing CAL. The reduction in PD was 3.25 ± 0.786 mm for group 1 and 3.29 ± 1.013 mm for group 2. In terms of CAL gain, group 1 recorded a value of 4.65 ± 1.387 mm, compared with 5.07 ± 1.631 mm in group 2. Both groups showed a noticeable increase in radiographic bone filler, with an average of 69.85 ± 17.773%. **Conclusions**: The two regeneration techniques were both effective for the treatment of deep intraosseous defects, encouraging more substantial filling compared with preoperative levels.

## 1. Introduction

Periodontitis is a multifactorial inflammatory disease produced by periodontopathogenic bacteria, described by a progressive loss of attachment resulting in a loss of teeth [1]. Its primary features include the loss of periodontal support tissue, the main manifestation of which is clinical attachment loss (CAL), bone loss assessed by radiographs, the presence of periodontal pockets, and gingival bleeding [2,3].

According to the American Academy of Periodontology (2008), the purpose of periodontal regeneration is to restore destroyed periodontal tissue and improve dental stability, reconstructing the dental bone lost in cases in which there are intraosseous defects [1]. The treatment aims to reduce the pathogenic flora, thereby preventing destructive processes and reconstructing the bone defects caused by the disease.

The objective of periodontal regeneration is to optimize the biological mechanisms of periodontal healing, based on the principle described by Melcher in 1976 [4]. The nature and magnitude of the regeneration depend on the colonization and healing of cells that are responsible for the growth and distinction of new tissues [5].

In 1982, Nyman and Gottlow published an animal experiment study in which they demonstrated the effect of the different periodontal tissues on the healing process, with cells of the periodontal tissue ligament being the only cells with the capacity to form a new insertion [6]. Based on the results obtained to support these two hypotheses, the principle of cell exclusion of guided tissue regeneration (GTR) appears [6]. That research presented the results from a histological study in humans using the GTR technique, in which they described the creation of new cement with the insertion of connective tissue fibers [6,7]. Founded on the outcomes of these analyses, periodontal regeneration has been achieved with different biological principles: lyophilized demineralized bone allograft [8,9], the combination of barrier membrane and bone graft and enamel matrix derivatives [10,11,12,13].

In the last decade, there has been a growing interest in surgical procedures with a less invasive approach [2]. Developments in incision design, flap type, and materials used for periodontal regeneration have gained special interest in order to improve the maintenance of the clot space and increase flap stability and reduce the resulting gingival recession as much as possible [2].

Harrel and Rees suggested minimally invasive surgery (MIS) with the goal of reducing surgical damage [14]. This approach includes a microscope or magnifying lenses and the use of microsurgical instruments and materials [2,15]. Cortellini and Tonetti, in 2007, proposed the treatment of isolated intraosseous defects with periodontal regeneration by developing the concept of a minimally invasive surgical technique (MIST) [16]. The foundations of this technique were the concepts previously established by Harrel and Rees and the application of papilla preservation techniques with a microsurgical approach [17,18,19,20].

Subsequently, an improvement on this procedure was made—the modification of the minimally invasive surgical technique (M-MIST). It was designed in order to decrease the capacity for surgical invasion, and its main objectives were as follows: minimize injury to the interdental tissue while keeping the adjacent soft tissues intact to improve tissue stability and to decrease patient morbidity [21].

The qualitative variables involved in the use of these regenerative surgical techniques in conjunction with complementary regenerative biomaterials are currently controversial. The use of Emdogain^®^, alone or in combination with a bone substitute, allows favorable results to be achieved in the reconstruction of intraosseous defects, although unanimity has not been found in the literature on the best regeneration strategy [15,22].

Derived from the enamel matrix, Emdogain (EMD^®^) was introduced in 1971 and has become one of the most researched dental products. It is a compound derived from the proteins of the enamel matrix, of which, 90% is composed of amelogenin of porcine origin [1].

In the 1990s, Hammarstrom, Sven Lindskog, and Leif Blomloff discovered that EMD (enamel matrix proteins) could be used as a biologic mediator capable of inducing periodontal regeneration [23,24,25,26].

In successive studies, it was observed that EMD could play a role in cementogenesis. It is deposited on the root surface of developing teeth prior to cement development [26]. The rationale for the use of EMD in the treatment of intraosseous defects is to mimic the biological processes that take place in odontogenesis [27]. EMD stimulates fibroblast proliferation, periodontal ligament growth, and osteoblast proliferation and differentiation. In addition, it plays a significant role in healing by promoting angiogenic activity [26].

However, the regenerative potential of EMD appears to be limited in certain clinical circumstances, due to the difficulty of maintaining space due to the viscous nature of its preparation. The combination of EMD with diverse types of graft materials has been proposed as an alternative to prevent flap collapse and to enhance the results obtained by EMD alone for the treatment of deep vertical bone defects [2]. This combination aims to enhance the synergistic effect of both materials. EMD promotes cell proliferation and the expression of growth factors, cytokines, and extracellular matrix and induces the apoptosis of epithelial cells. On the other hand, bone grafting would increase the inducing potential and act as a scaffold for bone regeneration [28].

The regeneration can also be affected by patient-related factors and intrinsic factors of the tooth [2]. In 2012, Parashis et al. recorded clinical parameters that influence the prognosis of regenerative treatment. They reported the importance of considering preoperative probing depth to determine the most predictable surgical technique and the negative influence of parameters such as plaque index, gingival inflammation, and smoking habit on clinical attachment gain [28]. Cortellini et al. published clinical studies that reflected the influence of the morphology and angle of the defect on the results of regenerative therapy and provided data on the relationship between pulp condition and periodontal health [29,30,31].

However, the results of the studies published so far do not show statistically significant differences between the use of EMD alone or in combination with bone grafts, due to heterogeneity in terms of follow-up length, study design, and surgical procedure. Therefore, although there are comparative studies, the evidence is limited [32].

### Objective

Assess the clinical and radiological efficacy of minimally invasive surgical techniques for the treatment of infraosseous defects in periodontal regeneration procedures, comparing the use of Emdogain® alone or mixing it with a xenograft of bovine origin (Bio-oss^®^), for a one-year follow-up period.

## 2. Materials and Methods

### 2.1. Design of This Study

This prospective multicenter randomized controlled clinical study was conducted between October 2019 and May 2022, as part of a master’s degree in Periodontics and Implants at the University of Seville. All the patients were informed and signed a consent form, and the study protocol was in accordance with the Biomedical Research Law and approved by the Institutional Ethics Committee belonging to the Virgen Macarena and Virgen del Rocío University Hospitals, with the internal code 0811-N-19.

The surgical procedures and the recording of clinical parameters were performed by a calibrated and experienced examiner. The patients and the second examiner were unaware of the experimental surgical procedure. Periodontal defects were assigned with a balanced randomization in a sealed envelope to one of the two experimental surgical procedures, allowing the concealment of the sequence and the existence of biases.

### 2.2. Sample Study Population

Forty patients aged between 25 and 76 years (mean: 50 ± 25) with periodontitis stage III or IV were selected. After basic periodontal treatment, all of them had three-walled or combined infraosseous defects with probing depths ≥ 6 mm.

Forty-eight bone defects were subjected to data analysis. As a result of the calculation of the sample size, 20 defects were treated with EMD (Emdogain®, Institute Straumann AG, Basel, Switzerland) (group 1) and 28 defects were treated with EMD and xenograft (Bio-oss®, Geistlich, Switzerland) (group 2).

#### 2.2.1. Inclusion Criteria

Patients were included based on the following criteria:

- Patients in good general health.
- Presence of at least one tooth with an angular bone defect with a PD and CAL ≥ 6 mm and an intraosseous component ≥ 3 mm, predominantly affecting the interproximal area.
- Non-smoking patients or smokers of less than 10 cigarettes/day.
- Plaque rate and bleeding rate ≤ 15%.
- Good endodontic condition of the teeth—healthy or with correct pulp treatment.

#### 2.2.2. Exclusion Criteria

- Patients with systemic diseases that may influence periodontal healing were excluded.
- Patients who could not commit to maintenance reviews were excluded.

### 2.3. Clinical Examination

All the clinical parameters were analyzed at baseline and at one year after surgery. Clinical measurements such as probing depth of the defect, recession, and level of clinical attachment were recorded at six sites of the tooth with a PCP-UNC 15 probe. The PD and CAL represented correspond to the measurements at the deepest point of the intraosseous defect of each tooth. Tooth mobility was also evaluated, as it is a factor that can influence the clinical outcomes of regenerative therapy.

**Figure 1:**
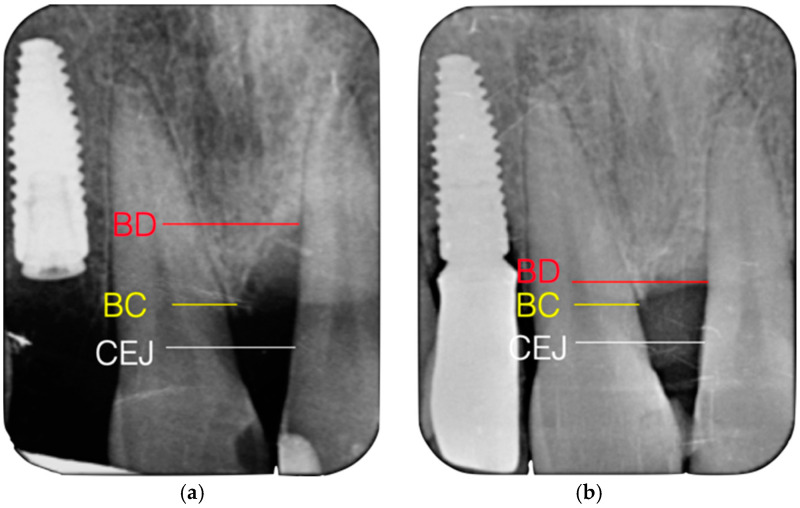
(**a**) Preoperative infraosseous defect. (**b**) Postoperative infraosseous defect.

Regarding intrasurgical measurements, the number of remaining bone walls and the infraosseous component of the defect were analyzed. We classified the infraosseous defects based on the number of residual bone walls, according to the system established [33]. In this study, only three-walled or combined defects were recorded. In addition, intrasurgical clinical measures were taken to define the infraosseous component of each defect: (1) distance from the amelocementary junction (CEJ) to the bottom of the defect (CEJ-BD); and (2) distance from the CEJ to the most coronal extension of the interproximal bony crest (CEJ-BC). The infraosseous component of the defect (INTRA) was defined as INTRA= (CEJ-BD) − (CEJ-BC) (Figure 1) [34].

### 2.4. Radiographic Evaluation

X-rays were taken using an individualized film dispositive with an XCP Ring parallelizer to always take the same projection and be able to compare the measurements. The digital radiographs were analyzed by a blind investigator using Image J software version 1.53a (Wayne rasband. National Institutes of Health, Kensington, MD, USA). The infraosseous component of the defect was also evaluated at the radiological level, performing the same measurements described above. The percentage of radiographic bone filling (RBF) was calculated, establishing a proportionality relationship between the baseline value and the post-surgical scores recorded with a minimum period of 12 months.

### 2.5. Follow-Up Period

Once the clinical and radiological diagnoses were made, all the patients underwent a basic periodontal treatment consisting of scaling and root planning and oral hygiene instructions. At 8 weeks, a periodontal re-evaluation was performed, in which, depending on the morphology of the infraosseous defect, probing depth, and mesiodistal width of the interproximal space, the most appropriate surgical approach was planned. To carry out this treatment, we used minimally invasive surgical techniques (MIST or M-MIST) together with the use of EMD, using it alone or together with a xenograft of bovine origin (BIO-OSS^®^), according to randomization.

After infiltrative local anesthesia, two incision techniques were selected according to the mesiodistal width of the interproximal space. The modified papilla-preservation technique (MPPT) was chosen in cases with an interdental area >2 mm, while the simplified papilla-preservation technique (SPPF) was applied when this width was <2 mm. The mucoperiosteal flap was designed according to the anatomy of the intraosseous defect. For defects that were limited only to the interproximal zone, an M-MIST design was made; on the other hand, if the defect extended to the lingual zone, the MIST technique was used.

**Figure 2:**
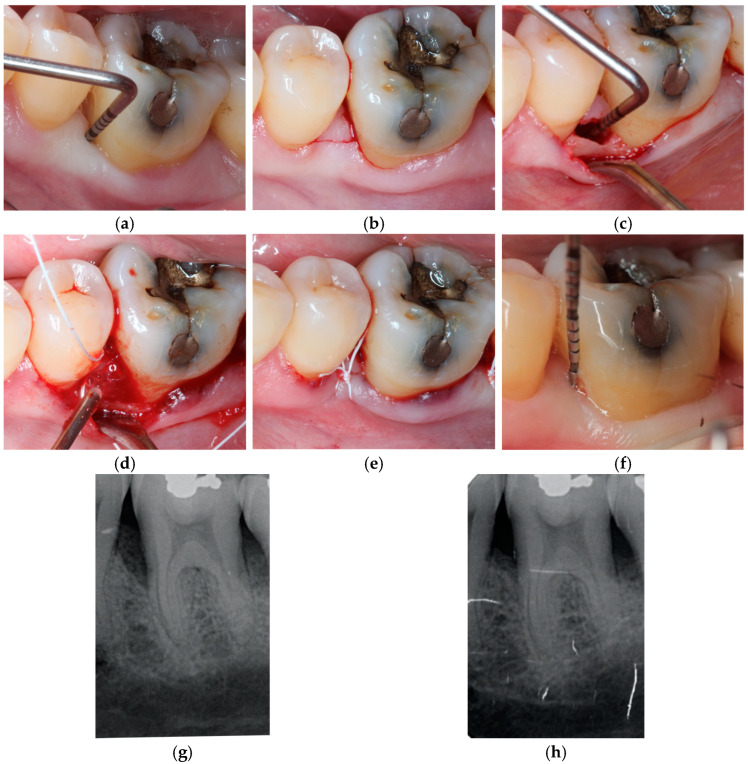
Case from group 1; defect treated with EMD. (**a**) Preoperative probing depth, (**b**) M-MIST incision, (**c**) intraosseous component defect, (**d**) EMDOGAIN application, (**e**) suture (**f**) postoperative probing depth, (**g**) preoperative radiograph, (**h**) postoperative radiograph at 1 year.

**Figure 3:**
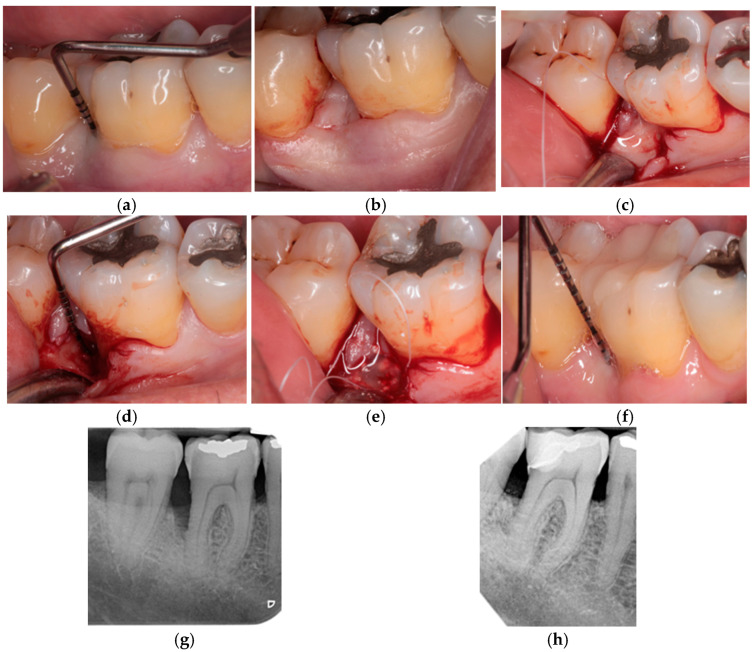
Case from group 2; defect treated with EMD + xenograft. (**a**) Preoperative probing depth, (**b**) M-MIST incision, (**c**) EDTA application, (**d**) intraosseous component defect, (**e**) EMDOGAIN and xenograft application (**f**) postoperative probing depth, (**g**) preoperative radiograph, (**h**) postoperative radiograph at 1 year.

Once the incision was made, the infraosseous defect was debrided, and a 24% EDTA acid was applied for two minutes for the removal of the remaining dentinal barrel. Subsequently, it was irrigated with saline solution and randomized in order to determine which material to use to fill the defect: EMD alone (group 1, Figure 2) or mixed with Bio-Oss^®^ (group 2, Figure 3).

Primary closure was achieved by means of modified vertical mattress pads, with a 4/0 polytetrafluoroethylene expanded suture. The postoperative recommendations included the administration of antibiotic treatment with amoxicillin 500 mg every 8 h for one week and analgesia with ibuprofen 600 mg three times daily. The application of 0.12% chlorhexidine twice a day was also advised, until mechanical removal of the plaque could be established.

A week later, the suture was removed. Subsequently, the patient was scheduled at six months and one year for a postoperative check-up in which periapical radiographs and analysis of clinical parameters were performed. During the first 6 weeks and thereafter, every three months during the first year of follow-up, they underwent periodontal support treatments, based on reinforcing oral hygiene and removing supragingival plaque, if necessary.

## 3. Results

### 3.1. Study Population and Analysis of Clinical Variables

A total of 40 patients were included in this prospective randomized clinical trial. Men and women aged between 25 and 76 (mean: 50 ± 25) were analyzed. Overall, 10% of the patients smoked <10 cigarettes per day. The result of the sample size calculation registered 48 defects. The data are expressed in mean ± SD for 20 defects (41.7%) of the 19 patients treated with EMD and for 28 defects (58.3%) of the 21 patients treated with EMD and xenograft. No intermittent participants were a part of this study, and there were no shortcomings in the data used for the analysis. The characteristics of the subjects are described in Table 1.

Table 2 summarizes the baseline and intrasurgical characteristics of intraosseous defects. The morphology of the defect in both groups was adapted in terms of the width of the defect and the number of remaining bone walls to regenerative procedures. Thirty-two of the defects were three-walled defects (66.7%); of which, seventeen defects (85%) were treated with EMD and fifteen defects (53.6%) with EMD and xenograft. Three combined defects (15%) were treated with EMD and thirteen defects (46.4%) with EMD and xenograft, out of a total of sixteen combined defects (33.3%).

Regarding the sites of the defect, twenty-eight were recorded in the mesial (58.3%), and twenty in the distal (41.7%) region. We analyzed the initial periodontal prognosis of the tooth in order to assess the influence of these intrinsic factors on the potential for regeneration. The initial prognosis was good in 32 defects (66.7%); 15 defects (31.3%) had a questionable prognosis; and only 1 defect (2.1%) had a poor prognosis. In group 1, 17 teeth (85%) had a good prognosis, and 3 (15%) had a questionable prognosis. On the other hand, in group 2, 15 teeth (53.6%) had a good prognosis, 12 had a questionable prognosis (42.9%), and 1 had a poor prognosis (3.6%).

### 3.2. Clinical and Radiographic Results

The mean initial PD was 8.35 ± 1329 mm, being 8.15 ± 1663 mm for group 1 and 8.50 ± 1036 mm for group 2, respectively. The mean baseline recession was 1.17 ± 1098 mm: for group 1, 1.05 ± 1099 mm and, for group 2, 1.25 ± 1.110 mm. Regarding the clinical level of insertion, a mean of 9.52 ± 1798 mm was initially recorded, 9.20 ± 1936 mm for group 1 and 9.75 ± 1691 mm for group 2. As for the intraosseous component, it was measured intraoperatively; the mean was 5.17 ± 1374 mm. The value for group 1 was 5.35 ± 1.694 mm and, for group 2, 5.04 ± 1.105 mm. There were no significant differences between the initial values of the variables examined.

The follow-up of this study was one year, with no differences in evolution regardless of the filling material used. Compared with the baseline, both treatment modalities resulted in statistically significant improvements in terms of a reduction in probing depth and as well as clinical attachment gain. The mean PD was reduced by 3.27 ± 0.917 mm, being 3.25 ± 0.786 mm for group 1 and 3.29 ± 1.013 mm for group 2. Regarding clinical insertion gain, the mean was 4.90 ± 1.533 mm: for group 1, 4.65 ± 1.387 mm and, for group 2, 5.07 ± 1.631 mm. Changes in the position of the gingival margin occurred between the beginning of treatment and one year of treatment in both groups, with a mean of 1.63 ± 1.248 mm. It did not reach statistical significance for group 1 (1.40 ± 1.231 mm) or group 2 (1.79 ± 1.258 mm), since the average increase in recession was 0.46 mm.

Bone changes were measured at the radiological level as a percentage of filling of the intraosseous component. Both groups had a gain in apparent radiographic bone filling, compared with the baseline, with the mean being 69.85 ± 17.773%. The percentage of radiographic bone filling was higher for group 1 (72.60 ± 16.19%) compared with group 2 (67.89 ± 18.79%), although the differences between the groups were not statistically significant (Table 3). An analysis of the cases was carried out in the case of patients who smoked, and no differences were observed in terms of the clinical variables analyzed. In relation to endodontic status, no significant differences were found between the groups. Only one case was recorded that required post-treatment endodontic treatment, without influencing the outcome of regeneration (Table 4).

## 4. Discussion

The present study demonstrates that the treatment of intraosseous defects with EMD alone or in combination with xenograft generates statistically significant improvements in terms of the reduction of probing depth and clinical insertion gain, during a one year follow up.

Studies such as that of Cortellini et al. [30] evaluated the efficacy of M-MIST alone or in combination with EMD or EMD/xenograft. All the three groups had a significant gain from baseline, with the percentage of RBF being 77 ± 19% for the M-MIST group, 71 ± 18% for the EMD group, and 78 ± 27% for the EMD/xenograft group. There were no statistically significant differences between the groups.

As described in the previous section, our results showed a reduction in probing depth from 3.25 ± 0.786 mm for group 1, 3.29 ± 1.013 mm for group 2, as well as a clinical insertion gain of 4.65 ± 1.387 mm for group 1 and 5.07 ± 1.631 mm for group 2. This was not statistically significant between the two groups. Regarding the change in the position of the gingival margin, it did not reach statistical significance between groups (being, for group 1, 0´35 mm and, for group 2, 0,54 mm). In both groups, there was an apparent percentage gain in filling of the intraosseous component, with a mean of 69.85 ± 17.773%. The RBF was superior for group 1 (72.60 ± 16.19%) compared with group 2 (67.89 ± 18.79%), although the differences between the groups were not statistically significant.

Zucchelli et al. [35] evaluated the adjuvant effect of bovine porous bone grafting (BPBM) in the treatment of intraosseous defects through a controlled clinical trial. Both procedures achieved clinically and statistically significant improvements in terms of clinical attachment gain, reduction in PD, and RBF. However, the use of BPBM in conjunction with EMD showed superiority over EMD in terms of CAL (5.8 ± 1.1 mm vs 4.9 ± 1.0 mm), depth of radiographic bone filling (5.3 ± 1.1 mm vs 4.3 ± 1.5 mm), and smaller increase in gingival recession (0.4 ± 0.6 mm vs 0.9 ± 0.5 mm).

Leonardis et al. [36] and Lekovic et al. [37] stated that the effect of combination therapy allowed for a greater reduction in PD and an increase in CAL, compared with treatment with EMD alone. In both studies, the maintenance of space by the xenograft could have contributed to the differences in defect filling, as it aids blood clot stability and isolates gingival epithelial cells and connective tissue from the defect area.

Lekovic et al. [37] conducted a randomized split-mouth clinical study to evaluate the clinical efficacy of EMD used alone or in combination with bovine porous bone grafting (BPBM) in the treatment of intraosseous defects. The results of that study confirm a greater reduction in PD in the EMD/BPBM group (3.43 ± 1.32 mm in the buccal and 3.36 ± 1.35 in the lingual), compared with the EMD group (1.91 ± 1.42 mm in the buccal and 1.85 ± 1.38 mmm in the lingual).

Zhang et al. [38] obtained better results with the use of a xenograft in regenerative therapy, stating that EMD is not the indicated material for the treatment of broad defects, due to the viscous nature of its formulation.

Similarly, Matarasso et al. [1] and Tsai et al. [39], in their systematic review and meta-analysis, provided a biological justification for the use of this combination in regenerative therapy. Since the xenograft stimulates the release of growth factors and cytokines, it establishes a synergistic relationship between both materials: the osteoconductive function of the xenograft by maintaining space and the influence of the EMD in the formation of root cement, ligament, and bone. Findings from this meta-analysis have shown better outcomes in defects treated with EMD and bone grafts than in defects treated with EMD alone, although the evidence to support this concept is lacking due to the variability of controlled clinical trials.

On the contrary, there is controversy about the benefit of the contribution of xenografting in improving EMD outcomes. Li et al. [32] conducted a meta-analysis to compare the clinical outcomes of EMD alone or in combination with multiple bone grafts. They supported the previous reasoning of the synergy between both materials and the improvement in the clinical evolution of the use of combination therapy in the treatment of deep vertical bone defects, although the results were not satisfactory. An evaluation of 11 studies over an 8-month follow-up period indicated that combination therapies produced better outcomes regarding increased defect filling and decreased gingival recession when compared with EMD alone. There were no differences observed in terms of PD reduction and CAL gain. On the other hand, the long-term benefits proved to be minimal, similar to the findings of our study. Therefore, further research is needed to confirm the superiority of one technique over another.

Likewise, Hasuike et al. [40], in their systematic review, evaluated the efficacy of both therapies and concluded that combined therapy only improves results with respect to radiographic bone filler.

The plaque index and bleeding index were low (less than 15%) at the beginning of this study and were maintained during the follow-up period, due to the fact that the patients were instructed in hygiene habits, and periodic periodontal maintenance was performed. According to the literature review, poor control of bacterial plaque is related to poor clinical outcomes [3].

Another factor that has been shown to significantly affect healing and the result of regenerative therapy is tobacco. Döri et al. [41] in their 10-year randomized clinical study observed that there was a loss of 2 mm of CAL after regenerative therapy in patients who smoked. Therefore, our study excluded those who smoked more than 10 cigarettes a day, with an average of 10% of our study patients being smokers, with no significant clinical differences observed among them.

Kornman [42] mentioned that the morphology of the defect plays an important role in the clinical outcomes of periodontal surgery, since it has been shown that clinical attachment gain is related to the number of remaining bone walls. However, in our study, the number of bone walls demonstrated a reduced impact on the results regardless of the technique used, as did the studies by Tonetti et al. [16] and Moreno et al. [43]. These studies also reinforced that the use of combination therapy allows the reduction and preservation of interproximal soft tissue in deep, isolated, and uncontained periodontal defects.

The endodontic stage and the dental prognosis have been considered relevant factors in the outcome of periodontal therapy. In our study, only one case required post-treatment endodontic treatment, which did not influence the outcome of regeneration. These data confirm that root canal treatment did not negatively affect the healing response and that the stability was retained, as stated in a clinical study by Cortellini and Tonneti [3].

Since no histological evaluation of the tissues was performed, no definitive statement can be made of the bonding nature of the regenerated tissues and root surface. Another limitation of this study is the sample size, which can influence the results. Studies with a bigger sample size must be performed to demonstrate the difference between the two modalities of treatment, although the results available in this study indicate that both of them are effective in improving the clinical parameters and the percentage of radiographic bone filling of the defects, as demonstrated in this study and other studies.

## 5. Conclusions

Despite its limitations, the present randomized clinical trial demonstrated that both regenerative approaches obtained clinical improvements. The results were not statistically significant regarding reduction in PD, clinical insertion gain, and percentage of RBF. However, group 1 demonstrated better results for radiographic bone filling than group 2.

To conclude, this study indicates that both regeneration techniques are effective for treating deep intraosseous defects, promoting the filling of the defect compared with pre-surgical levels. However, more randomized controlled clinical trials are needed to confirm the clinical benefits of using EMD alone or in combination with bone grafts to promote periodontal tissue regeneration.

## Figures and Tables

**Table 1:** Sample size.

Demographic Data (40 Patients)	Median ± SD
No. of defects	48
Age (years, media ± DE)	50 ± 25
Men/women	20/28
Smoking habit (%)	10

**Table 2:** Clinical data.

Variable	Biomaterial		Total
	EMD (Group 1)	EMD + Xenograft (Group 2)	
Frequency (No.)/Percentage (%)	20 (41.7%)	28 (58.3%)	48 (100%)
Initial stage			
Good	17 (85%)	15 (53.6%)	32 (66.7%)
Questionable	3 (15%)	12 (42.9%)	15 (31.5%)
Poor	0	1 (3.6%)	1 (2.15)
Site			
Mesial	9 (45%)	19 (67.9%)	28 (58.3%)
Distal	11 (55%)	9 (32.1%)	20 (41.7%)
Anatomy			
3-wall defect	17 (85%)	15 (53.6%)	32 (66.7%)
Combined	3 (15%)	13 (46.4%)	16 (33.3%)

**Table 3:** Clinical parameters.

	Follow-Up Period					
	Base			+12 Months		
	Group 1	Group 2	Media	Group 1	Group 2	Media
PD (mm)	8.15 ± 1.663	8.50 ± 1.036	8.35 ± 1.329	3.25 ± 0.786	3.29 ± 1.013	3.27 ± 0.917
REC (mm)	1.05 ± 1.099	1.25 ± 1.110	1.17 ± 1.098	1.40 ± 1.231	1.79 ± 1.258	1.63 ± 1.248
CAL (mm)	9.20 ± 1.936	9.75 ± 1.691	9.52 ± 1.798	4.65 ± 1.387	5.07 ± 1.631	4.90 ± 1.533
Infraosseous component (mm)	5.35 ± 1.694	5.04 ± 1.105	5.17 ± 1.374			
% of filling				72.60 ± 16.19	67.89 ± 18.79	69.85 ± 17.73

**Table 4:** Endodontic status.

Variable	Treatments		
Frequency (No.)/Percentage (%)	EMD	EMD + Xenograft	Total
Vital	19 (95%)	25 (89.3%)	44 (91.7%)
Previous endodontic treatment	0	3 (10.7%)	3 (6.3%)
Later endodontic treatment	1 (5%)	0	1 (2.1%)

## Data Availability

The data of our research can be consulted by email to brios@us.es.
